# Supplementary material for: Polymorphism and Ligand Binding Modulate Fast Dynamics of Human Telomeric G-Quadruplexes
Source: Int J Mol Sci. 2023 Feb 21;24(5):4280. doi: 10.3390/ijms24054280 (PMC10001961; doi:10.3390/ijms24054280)
Supplement: Supplementary file 1 [file ijms-24-04280-s001.zip › ijms-2160939-supplementary.pdf]

Supplementary Materials: Polymorphism and Ligand Binding Modulate Fast Dynamics of Human Telomeric G-Quadruplexes

Luca Bertini [1,†], Valeria Libera [1,2,†], Francesca Ripanti [1,*], Francesca Natali [3], Marco Paolantoni [4], Andrea Orecchini [1,2], Alessandro Nucara [5], Caterina Petrillo[1], Lucia Comez [2,*], and Alessandro Paciaroni [1]

1. Infrared experiments

The vibrational band ascribed to the guanine N7-C8-H bending mode when the N7 atom is involved in Hoogsteen hydrogen bonds is a clear marker of the G4 structure formation. DRIFT spectra in this spectral range are reported in Figure S1, in order to confirm the presence of G4 structures also in the case of poor-water samples.
From IR measurements, information on the water contribution can be obtained. In particular, the OH stretching band of water can be reproduced considering three distinct contributions [1]. The first one at around 3300 cm^{-1} is representative of the so-called connective water, where the OH oscillators are phase-correlated with oscillators of the closest molecules, connected by stronger H-bonds; the second one at around 3470 cm^{-1} is associated to close water structures, where H-bonds are partially distorted and the phase correlation among the oscillators is lost; and the last one located at higher wavenumbers around 3610 cm^{-1} is due free OH groups [1–3]. The ATR-FTIR spectra in the OH stretching region, reported in Figure S2, evidence that the overall distribution is shifted at lower wavenumbers for the Tel22 powders with Na^+ cations. This suggests that the water molecules localized around the antiparallel conformation tend to form relatively stronger hydrogen bonds than those surrounding the parallel one.

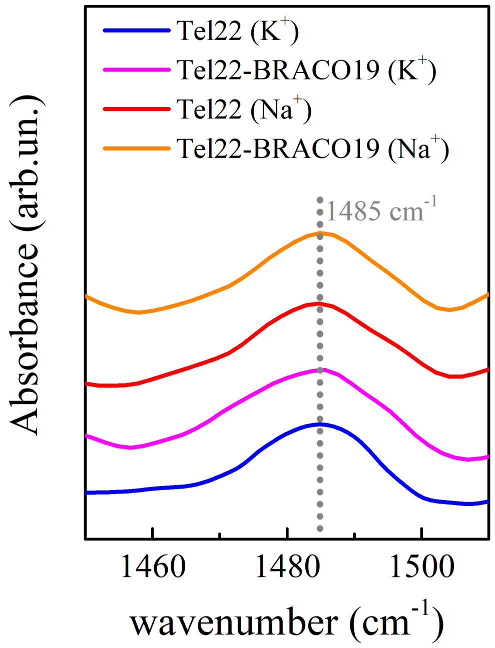

Figure S1. DRIFT spectra of Tel22 (K^+), Tel22 (Na^+), Tel22-BRACO19 (K^+), and Tel22-BRACO19 (Na^+) in the [1450 - 1510] cm^{-1} where the peak due to the guanine N7C8H bending vibration when the N7 atom is involved in Hoogsteen hydrogen bonds [4] is present.

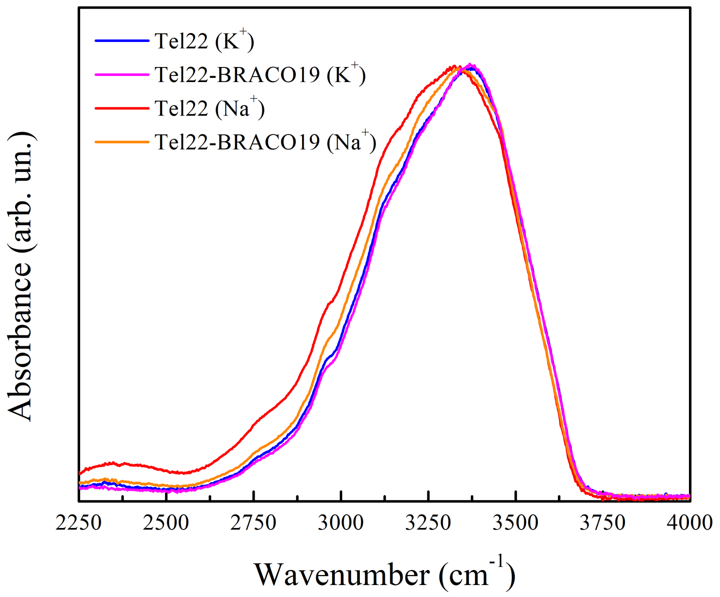

Figure S2. FTIR-ATR spectra of Tel22 (K^+), Tel22 (Na^+), Tel22-BRACO19 (K^+), and Tel22-BRACO19 (Na^+) in the high wavenumber region, mainly ascribed to the OH stretching vibrations.

2. Circular Dichroism

Circular Dichroism (CD) is a powerful and well established technique to determine the secondary structure of G4 structures. In Figure S3 we report the CD spectra of Tel22 alone and Tel22-BRACO19 complex in diluted conditions both in K^+ and Na^+ ionic environments. The presence of different ions clearly affects the conformation of Tel22. Moreover, complexation with BRACO19 results in a prevalently antiparallel topology in both K^+ and Na^+ cases.

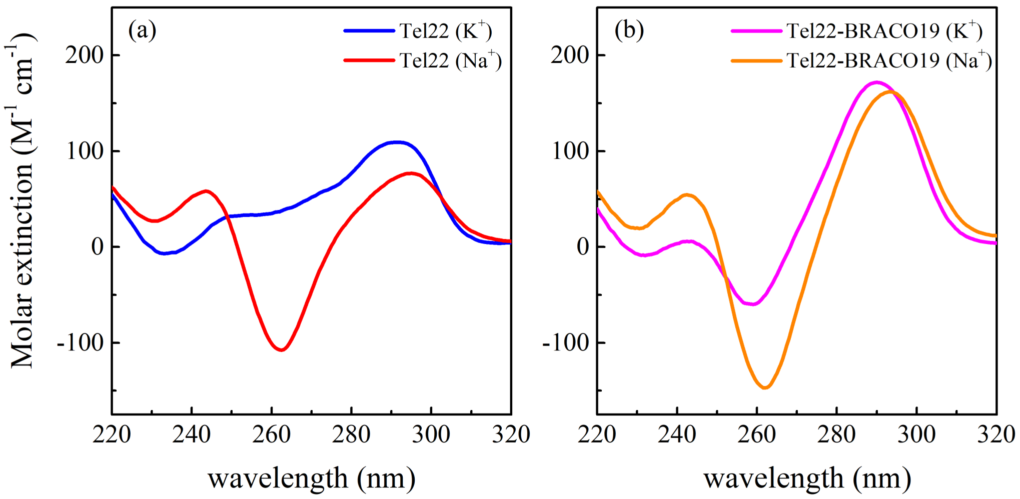

Figure S3. CD spectra in dilute solutions of: (a) Tel22 (K^+) and Tel22 (Na^+); (b) Tel22-BRACO19 (K^+) and Tel22-BRACO19 (Na^+). G4 concentration is equal to 40 μM.

References

1. Freda, M.; Piluso, A.; Santucci, A.; Sassi, P. Transmittance Fourier transform infrared spectra of liquid water in the whole mid-infrared region: temperature dependence and structural analysis. *Applied Spectroscopy* **2005**, *59*, 1155–1159.
2. Gallina, M.E.; Sassi, P.; Paolantoni, M.; Morresi, A.; Cataliotti, R.S. Vibrational analysis of molecular interactions in aqueous glucose solutions. Temperature and concentration effects. *The Journal of Physical Chemistry B* **2006**, *110*, 8856–8864.

3. Libera, V.; Bianchi, F.; Rossi, B.; D'Amico, F.; Masciovecchio, C.; Petrillo, C.; Sacchetti, F.; Paciaroni, A.; Comez, L. Solvent vibrations as a proxy of the telomere G-quadruplex rearrangements across thermal unfolding. *International Journal of Molecular Sciences* **2022**, *23*, 5123.
4. Guzmán, M.R.; Liquier, J.; Brahmachari, S.; Taillandier, E. Characterization of parallel and antiparallel G-tetraplex structures by vibrational spectroscopy. *Spectrochimica Acta Part A: Molecular and Biomolecular Spectroscopy* **2006**, *64*, 495–503.
